# Evaluation of the Policy Effect of China’s Environmental Interview System for Effective Air Quality Governance

**DOI:** 10.3390/ijerph18179006

**Published:** 2021-08-26

**Authors:** Xue Jin, Ussif Rashid Sumaila, Kedong Yin, Zhichao Qi

**Affiliations:** 1 School of Economics, Ocean University of China, Qingdao 266100, China; jinxue@ouc.edu.cn (X.J.); jx8212@ouc.edu.cn (Z.Q.); 2 Institute for the Oceans and Fisheries, University of British Columbia, 2202 Main Mall, Vancouver, BC V6T 1Z4, Canada; r.sumaila@oceans.ubc.ca; 3 Ocean Development Research Institute, Major Research Base of Humanities and Social Sciences of Ministry of Education, Ocean University of China, Qingdao 266100, China; 4 School of Public Policy and Global Affairs, University of British Columbia, 2202 Main Mall, Vancouver, BC V6T 1Z4, Canada; 5 Institute for Environment and Development (LESTARI), Universiti Kebangsaan Malaysia, Bangi 43600, Malaysia; 6 Institute of Marine Economy and Management, Shandong University of Finance and Economics, Jinan 250014, China; 7 School of Management Science and Engineering, Shandong University of Finance and Economics, Jinan 250014, China

**Keywords:** environmental interview, air governance, dynamic panel model, sharp RD

## Abstract

The Ministry of Ecology and Environment of the People’s Republic of China formally proposed an environmental interview system in May 2014, which applies pressure on local governments to fulfill their responsibility toward environmental protection by conducting face-to-face public interviews with their officials. In this paper, 48 cities that were publicly interviewed from 2014–2020 were considered the experimental group and 48 cities surrounding them were the control group. First, the dynamic panel model is applied to initially determine the effect of the policy. Then, a regression discontinuity method (Sharp RD) is used to analyze the short-term and long-term effects and compare the reasons for the differences observed among the estimates of various types of samples. Finally, a series of robustness tests were also conducted. The results show that the environmental interview system can improve air quality. However, because an emergency short-term local governance system exists at present, the governance effect is not long-term and, therefore, not sustainable. Therefore, it suggests that the government should continue to improve the environmental interview system, establish an optimal environmental protection incentive mechanism, and encourage local governments to implement environmental protection policies effectively in the long term. The results of the research are of great significance to the environmental impact assessment system of the world, especially in countries with similar economic systems, which are facing a trade-off between economic growth and environmental sustainability.

## 1. Introduction

With the continuous development of the global economy, a healthy ecological environment has become a growing demand for a better life, especially in terms of air quality that has been closely monitored. Since the 1950s, London has suffered from dense fogs; in 1956, the United Kingdom promulgated the Clean Air Act, becoming the world’s first country to enact an air pollution prevention and control bill. Following the implementation of this act, governments around the world have formulated similar air pollution prevention and control bills and policies. Zhang et al. [1] pointed out that less than 1% of China’s large cities can meet the international air quality standards. This shows that air pollution has become a persistent problem affecting the construction of an ecological civilization [2], posing a great threat to public health and daily life [3]. Due to the growing need for air pollution treatment, many scholars working in the fields of meteorology [4,5,6,7], physics [8,9,10,11], and chemistry [12,13,14,15] have studied the effects of air pollution. Additionally, many economists have studied the relationship between the economy and air pollution—the direct and indirect effects of air pollution on the economy. The research includes the impact of economic development on air pollution [16,17,18] and the economic loss caused by air pollution [19,20,21].

By applying a regression discontinuity (RD) design, Chay et al. [22] used the Clean Air Act (and its amendments enacted in 1977 in the United States) as the driving variable and total suspended particulate (TSP) matter as the dependent variable to prove that air quality improved following the enactment of the act. This study was one of the earlier studies to assess the effect of environmental policy implementation from a quantitative perspective. Subsequently, in recent years, the system of policy evaluation methods has been continuously improved upon, and the number of relevant policy-effect evaluation studies has increased as well. Hering et al. [23] used an instrumental variable approach to study the role of China’s environmental regulations on export trade. Antimiani et al. [24] studied the impact of an EU policy on reducing greenhouse gas emissions. Zhang et al. [25] used an RD design to assess the impact of central regulation on chemical oxygen demand (COD) emissions of an industrial firm through the National Specially Monitored Firms program. Nabernegg et al. [26] combined a computable general equilibrium model with multi-regional input-output models to study the effectiveness of carbon reduction policies in international supply chains and deduced that the policies reduced consumption-based carbon emissions. Wang et al. [27] studied the impact of China’s environmental tax policy on environmental efficiency in different regions using provincial environmentally extended input-output tables and a frontier-based optimization model. Zhang et al. [28] measured the efficiency of the implementation of the policy using the data envelopment analysis–slacks-based measure model to analyze the emissions trading system in China and used the differences-in-differences (DID) model to examine the efficiency of the policy in terms of its environmental impact. Chen et al. [29] used the DID model along with multiple regression models to study the carbon reduction effects of China’s pilot emissions trading scheme and the impact of the policy on national and regional-level impact pathways. The results show that the policy reduced total carbon emissions by 13.39%, with an increasing reduction observed year by year.

**Figure 1:**
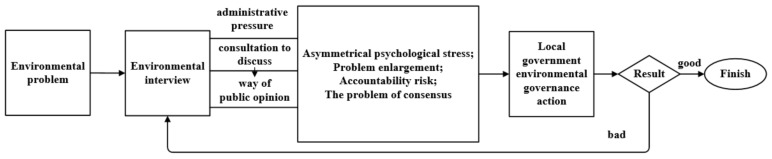
Influence mechanism and process of central environmental consultation on local governments.

Environmental protection interview refers to an administrative measure taken by the Ministry of Environmental Protection to interview the responsible persons of local governments and relevant departments who fail to perform their environmental protection duties or fail to perform their duties in place, conduct warning talks, point out relevant problems, put forward rectification requirements and urge the rectification in place according to law [30]. The process of the environmental interview system is shown in Figure 1. The interview system, which is an innovative system in China, is also an environmental policy tool. It originated from the “coffee drinking” system in Hong Kong, the name of which is an unofficial description of a system in Hong Kong and it mainly occurs for reviewing the taxes of tax subjects by the Inland Revenue Department. Similar to the ‘tax interview system’ in the United States and the ‘forgiveness system’ in Europe and the United States. This mode of negotiation between the administrative subjects and their counterparts that need no mandatory court order was widely used in China and it became the administrative interview system. Research is mainly derived from theoretical studies that have legal and administrative perspectives, such as the concept, content, legal attributes, mechanism of action, and institutional improvement of the interview system. Studies carried out from the perspective of economics and policy evaluation are rare. In specific research, such as Pranis [31], tax authorities are empowered to negotiate and mediate with taxpayers if problems and disputes arise from taxation under legal conditions. Meyercord [32] pointed out that the Internal Revenue Service (IRS) in the US is authorized by law to reach concessionary agreements on tax arrears when certain conditions are met. Hanlon [33] argued that a tax reconciliation system can do both—eliminate dissatisfaction between tax collectors and taxpayers and increase government revenue. Using a sample of 619 cartels, Choi [34] found that leniency regimes are ineffective in the short run; however, they increase the probability of collapse eventually, thereby narrowing the time interval. Shi et al. [35] used the RD model to evaluate the effect of the environmental interview system on air pollution, and the empirical results show that the policy has a significant effect on air pollution only when the authorities of cities are interviewed regarding air pollution issues. Wu et al. [36] used the DID model to test the relationship between the environmental interview system and the efficiency of environmental governance of a local government. 

In summary, awareness of the importance of the environment is gradually increasing, and the environmental interview system is innovative. Only a few similar systems and theories of interviews can be found, and the quantitative evaluation of the system is also at a preliminary stage. Therefore, from the perspective of policy evaluation, it is necessary to meticulously, comprehensively, and quantitatively assess the effect of the interview system of the Ministry of Environmental Protection (MEP) on air pollution governance.

This paper evaluates the policy effect of China’s environmental interview system from the perspective of air quality governance and provides an in-depth analysis of its influencing mechanism. The innovative contributions of this paper include the following: (1) Existing studies have used the date of the interview as the discontinuity position, ignoring the time-lag of the interview system; therefore, we adopted a city-by-city approach to determine the point of improvement that was further used to deduce the real discontinuity position; (2) A long-term effect estimation method was proposed with weeks as the time interval; the period was extended seven times to meet the sample size required to determine whether the environmental talks were long-term; (3) An air quality data authenticity analysis method was modified to rule out the possibility of data falsification that can otherwise lead to unreal policy effects.

The remaining part of the paper is structured as follows: Section 2 describes the study area, variables, data sources, and methods. Section 3 illustrates the empirical results of least squares dummy variable analysis, determines the point in time when the policy works, and evaluates the short- and long-term effects of the environmental interviewing system. Section 4 details the robustness check to understand the reliability of the assessment results by analyzing the control group, the sensitivity of the bandwidth, and the validity of the air quality data. Section 5 discusses and suggests the future issues be addressed. The conclusions and recommendations are presented in Section 6.

## 2. Materials and Methods

### 2.1. Study Area and Data Sources

**Figure 2:**
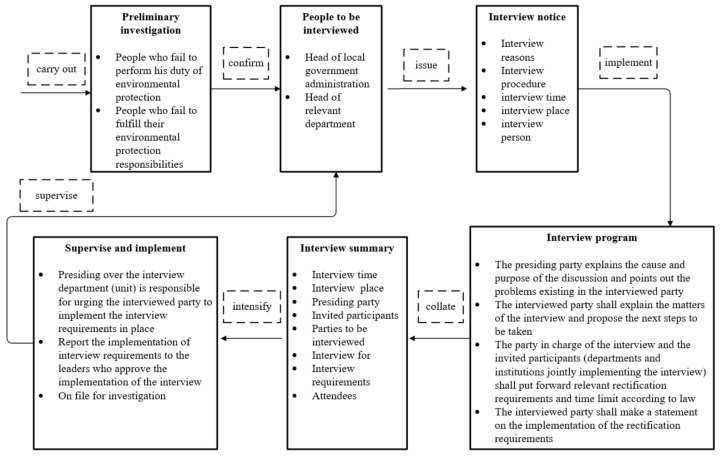
Structure and process of environmental interview system.

Through an extensive review of relevant data, a list of 50 cities (the word ‘city’ is used instead when it comes to a city where the city officials or other representatives are interviewed) (districts and counties) that were interviewed from 2014 to 2020 was compiled. The process of the environmental interview system is shown in Figure 2. Owing to the non-availability of air quality data for cities such as Hengyang and Liupanshui, 48 samples were used to carry out an empirical analysis, 37 of which had air quality problems. 

Concurrently, we also considered the same administrative jurisdiction and excluded the influence of differences in natural environmental conditions and environmental protection policies as much as possible. We selected cities with the most similar air quality changes and determined the closest cities as the control group by comparing the air quality trends observed over six months before the interviews with the experimental group were conducted (see Table A1).

In this paper, the daily data of air quality index (AQI) and the corresponding evaluation indices of sulfur dioxide (SO_2_), nitrogen dioxide (NO_2_); PM10, PM2.5, ozone (O_3_); and carbon monoxide (CO) specified in the ambient air quality standards are considered as dependent variables. Raw data for each indicator comes from the Peoples Republic of China’s State Environmental Protection Administration website.

There are three independent variables: (1) time [T] before and after the dummy interview variable, which is also called the driver variable that determines whether the policy is working or not; (2) the number of days [d] with time-lag for each interviewed city, which determines where the air quality improves; and (3) the time variable polynomial [f(d)] from the date of the interview to reflect the effect of gradual implementation of the environmental interview system.

The control variables consider the weather conditions to determine whether the sample is a heating city, whether it is in the process of heating, and whether it is a working day. This study helps to estimate the net policy effect of the environmental interview system by including control variables in the model, thus excluding the effects of weather, time, and geographic factors on air governance. The symbols for each category of variables and their specific meanings are shown in Table 1.

### 2.2. Methodology

#### 2.2.1. Dynamic Panel Model

Weather conditions affect air quality, and as such including it as a variable in the model allows for better identification of the net effect of the environmental covenant system. Concurrently, domestic emissions in heating cities during the heating period will be significantly higher than those in non-heating cities. Most industries have weekend shutdowns and, therefore, it is also necessary to consider the scenarios of dummy variables—i.e., whether they are in heating cities, whether they are cities that are being heated, and whether it is a working day. Combining these factors, a model is designed as follows

(1) $$Y_{ij}=\alpha_{0}+\alpha_{1}T_{ij}+X_{ij}+u\times I+v\times J+w\times K+\gamma_{i}+f(d)+\varepsilon_{ij}$$

where

$Y_{ij}$ is the air quality variable (AQI, PM_2.5_, PM_10_, SO_2_, NO_2_, CO, or O_3_) for the city *i* at date *j*,

$T_{ij}$ is a dummy variable for the interview status of the city,

$X_{ij}$ is a set of weather control variables, 

*I*, *J*, and *K* are dummy variables for *i* to indicate whether the city is a heating city, whether it is in a heating period, and whether it is a working day, respectively,

$\gamma_{i}$ is an individual fixed term, i.e., there is inherent variability across cities, 

*d* is used to denote the number of days until the day of the MEP interview,

*f(d)* is a polynomial about d (the air improvement caused by the gradual progress of pollution prevention and control efforts), 

$\varepsilon_{ij}$ is the random disturbance term,

$\alpha_{1}$ represents the difference in air quality before and after the environmental interview, which is the focus of the policy evaluation,

$\alpha_{0}$ represents the constant term,

*u*, *v*, and *w* are the coefficient values of each variable.

Considering that the pollutants emitted to the atmosphere are not immediately transferred or transformed but slowly accumulated in the air—i.e., there is a cumulative effect—the air quality of the day is often affected by the air quality of the previous few days. Therefore, a lagged order of the dependent variable is added to the model, and the cumulative effects of air pollution, control variables, and time-individual fixed effects are considered to obtain the model as shown in Equation (2).

(2) $$Y_{ij}=\sum_{s=1}^{p}Y_{i,j-s}+\;\alpha_{0}+\alpha_{1}T_{ij}+X_{ij}+u\times I+v\times J+\;w\times K+\gamma_{i}+f(d)+\varepsilon_{ij}$$

where $Y_{i,j-s}$ is the lagged value of $Y_{ij}$, its p is the lagged order, the lagged order is related to the practice sequence *j*, and the other variables have the same meaning as in Equation (1). Equation (2) contains both time and cross-sectional dimensions. The lag terms of the explained variables are added on the right side of the equation and, therefore, it is called the dynamic panel model.

#### 2.2.2. Regression Discontinuity Model

There are three main sources of air quality improvement: (1) Group effect: individual heterogeneity, which means that different cities respond differently to environmental interviews, which is closely related to their weather conditions, geographic location, industrial structure, and level of economic development; (2) Time effect: regardless of whether they are interviewed or not, air quality changes over time; (3) Policy treatment effect: the environmental interview system leads to the improvement of air quality.

A sample of four weeks before and after the interview was used as the object of study; the time window was set to be small and other factors affecting air quality were not easy to change substantially. Thus, the RD model can better solve the endogeneity problem caused by the omitted variables. In the dynamic panel model, the right side of the equation contains the lagged order of the explained variables. It is not possible to solve the endogeneity problem using the static-dynamic panel model estimation method and the LSDV estimation method alone and, therefore, the RD method should also be used for the analysis.

Viard et al. [37] set up the model as an explicit regression discontinuity (sharp RD) as given in Equation (3)

(3) $$Y_{ij}=\alpha_{0}+\alpha_{1}T_{ij}+\alpha_{3}\times f(d)+X_{ij}+u\times I+v\times J+\;w\times K+\gamma_{i}+\varepsilon_{ij}$$

The meaning of each of these variables is the same as in Equation (1).

$Y_{ij}$ is the air quality variable (AQI, PM_2.5_, PM_10_, SO_2_, NO_2_, CO, or O_3_) for the city *i* at date *j*,

$T_{ij}$ is a dummy variable for interview status of the city,

$X_{ij}$ is a set of weather control variables, 

*I*, *J*, and *K* are dummy variables for *i* to indicate whether the city is a heating city, whether it is in a heating period, and whether it is a working day, respectively,

$\gamma_{i}$ is an individual fixed term, i.e., there is inherent variability across cities, 

*d* is used to denote the number of days until the day of the MEP interview,

*f*(*d*) is a polynomial about *d* (the air improvement caused by the gradual progress of pollution prevention and control efforts), 

$\varepsilon_{ij}$ is the random disturbance term,

$\alpha_{1}$ represents the difference in air quality before and after the environmental interview, which is the focus of the policy evaluation.

$\alpha_{0}$ represents the constant term,

*u*, *v*, and *w* are the coefficient values of each variable.

## 3. Empirical Results and Analysis

### 3.1. Results of the Least Squares Dummy Variable Analysis

Since the cross-sectional number (m) used in this study is small (m = 48) and the time dimension (*n*) is large (*n* = 119), the long panel can be estimated directly using the least squares dummy variable (LSDV) method. The regression results are shown in Table 2.

According to the above table, air quality significantly improved after the interview—i.e., the interview dummy variables of AQI, PM_2.5_, PM_10_, SO_2_, NO_2_, CO, and O_3_ were all negative at the 1% significance level—suggesting that the air quality indicators significantly reduced after the interview, thereby suggesting that the environmental interview system improved air quality.

The specific paths of the influence of weather factors on air quality were as follows:

(1) For the two dummy variables of rainfall and snowfall, the LSDV regression results show that rainfall significantly improved air quality more than snowfall. In terms of the effect of rainfall, the concentrations of pollutants such as AQI, PM_2.5_, PM_10_, SO_2_, NO_2_, and O_3_ were observed to be significantly negative, whereas CO was not. In terms of the effect of snowfall on the concentrations of pollutants, only SO_2_ was observed to be significantly negative while the remaining pollutants were negative too but not significantly so because the solubility of rainwater is better than that of snow; PM_2.5_, PM_10_, SO_2_, NO_2_, and CO dissolve in rainwater easily compared to snow and, therefore, the increase in precipitation frequency and rainfall results in increased concentrations of pollutants being dissolved, thereby reducing the concentration of pollutants in the air. Interestingly, SO_2_ was significantly lower during snowy weather because people will avoid using private cars and taxis to ensure travel safety resulting in lower vehicular exhaust emissions than usual.
(2) Maximum temperature was positively and significantly correlated with values of AQI, PM_2.5_, PM_10_, NO_2_, CO, and O_3_ values, and it was positive but not significant for SO_2_. This means that the higher the maximum temperature, the greater the concentration of pollutants (AQI, PM_2.5_, PM_10_, SO_2_, NO_2_, CO, and O_3_), and the higher the degree of pollution. Elevation can improve the efficiency of pollutant decomposition and conversion, thereby reducing the concentrations of pollutants. However, after an in-depth analysis, we found that the highest temperature of the day was often accompanied by sunlight, temperature, and time; generally, the highest temperature occurs in the middle of the day at about 14:00 h. Under these circumstances, the lighting conditions were better, and SO_2_ and NO_2_ encounter light and heat to undergo chemical reactions, which result in the formation of gaseous multi-oxides. Correspondingly, the concentration of pollutants in the air will not be reduced. In addition, as daytime is the most active time for humans, this is the time when industrial, residential, and automobile, emissions are at their peak. At the highest temperature, sufficient light accelerates the generation of pollutants and their derivatives, whereas from morning to noon, accumulation is at its peak. The higher the maximum temperature, the more the amount of SO_2_ and NO_2_ pollutants released into the air, increasing air pollution.

The minimum temperature has a negative and significant correlation with pollutants as the mechanism is similar but with a contrasting outcome to that of the maximum temperature.

(3) Maximum wind speed has a significantly negative correlation with all the pollutants, which means that the higher the wind speed, the lower the concentration of pollutants in the air. This is because, for a city (district, county), under certain conditions of pollutant concentration, the atmospheric diffusion capacity is related to the concentration of pollutants in the air—i.e., the greater the wind speed—the more the diffusion and transportation of pollutants.

### 3.2. Determination of Discontinuity Location

An important prerequisite for an RD to be established is the existence of an unambiguous discontinuity. If the day of the interview is used as the time discontinuity, it is less likely that environmental (air) control measures were implemented in the area on the day of the interview. 

Since the implementation of environmental improvement actions by local governments should take place after the interviews and the pace of implementation varies from city to city, it is necessary to identify the point at which the air quality improves on a case-by-case basis and use this as the time discontinuity. The results are shown in Table 3. The numbers (0, 1, and 2) represent the day of the interview, e.g., 1 represents one day after the interview, and 2 represents two days after the interview. The significance represents whether there is a significant improvement at a given point. Some of the results show deterioration, which is an exceptional situation; the day-by-day estimates are not very good. The results show that the air quality of most cities improved significantly within 10 days of the interviews, indicating that the government had taken measures before submitting the corrective action plan and that the environmental interview system had an ‘immediate effect’.

### 3.3. Analysis of Short-Term Effects

To satisfy the condition that the sample is near the threshold, a short-term effect is estimated using data of four weeks before and four weeks after the discontinuity. A sample size of 2688 satisfied the sample size requirement (using the model according to Equation (3)). The order of f(d) was determined by substituting orders 1 through 4 into the model regression, comparing Akaike’s information criteria and Bayesian information criteria indices, and selecting from the first- and second-order polynomial equations with the best fit. The results of the RD estimation to deduce whether the interviews improved air quality are shown in Table 4 wherein the AQI, PM_2.5_, PM_10_, and NO_2_ concentrations are significantly negative, indicating that the local governments have taken active and effective measures to improve air quality after the environmental interviews. The estimated results for SO_2_, CO, and O_3_ are negative but not significant. This indicates that after the environmental protection interviews, local governments are likely to focus on the assessment of PM_2.5_ and PM_10_ and, therefore, the concentrations of these two pollutants will be significantly reduced. Although SO_2_ has been used as an assessment indicator for a long time, the governments are likely to take measures, such as the implementation of the desulfurization reform of coal-fired boilers and the elimination of yellow-label vehicles, after the interviews. However, probably due to the slow process of action, the improvement did not occur during the period of this study. For other pollutants that are not assessed or do not gain high attention (e.g., O_3_), or if the formation mechanisms of secondary pollutants are complicated, no improvement measures are taken even after the interview.

**Figure 3:**
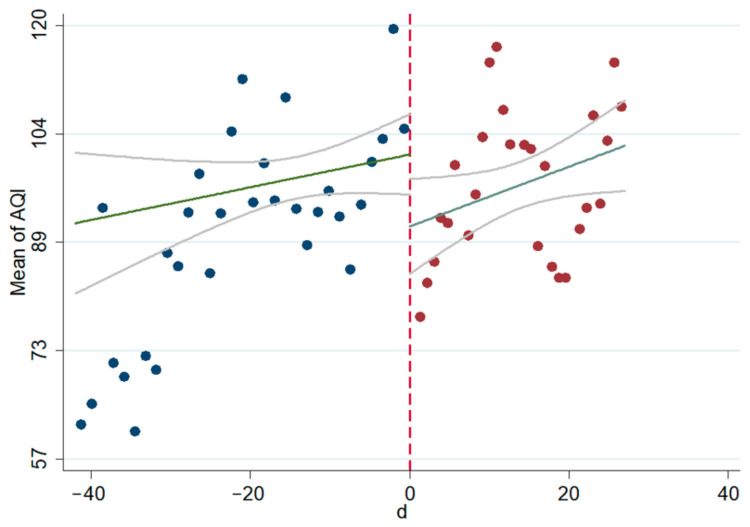
Scatter diagram of AQI before and after the interviews (linear).

**Figure 4:**
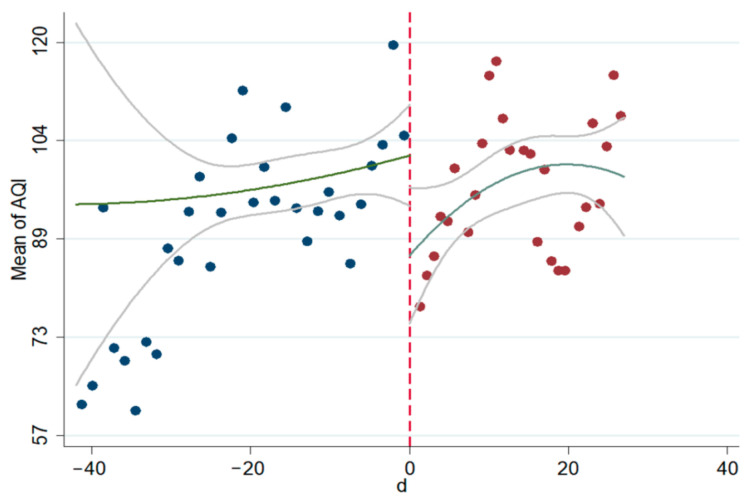
Scatter diagram of AQI before and after the interviews (nonlinear).

**Figure 5:**
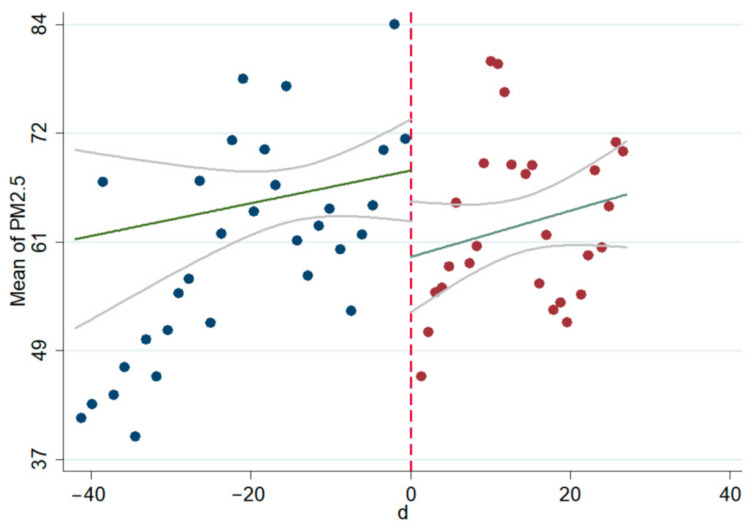
Scatter diagram of PM_2.5_ before and after the interviews (linear).

**Figure 6:**
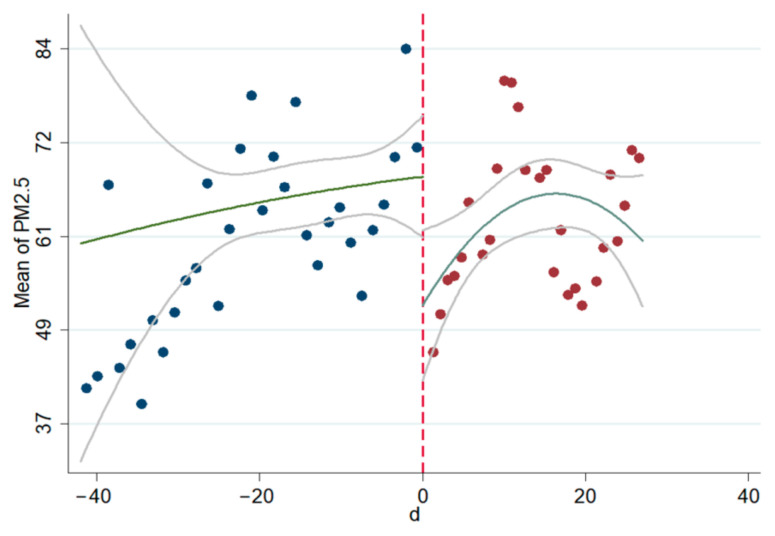
Scatter diagram of PM_2.5_ before and after the interviews (nonlinear).

**Figure 7:**
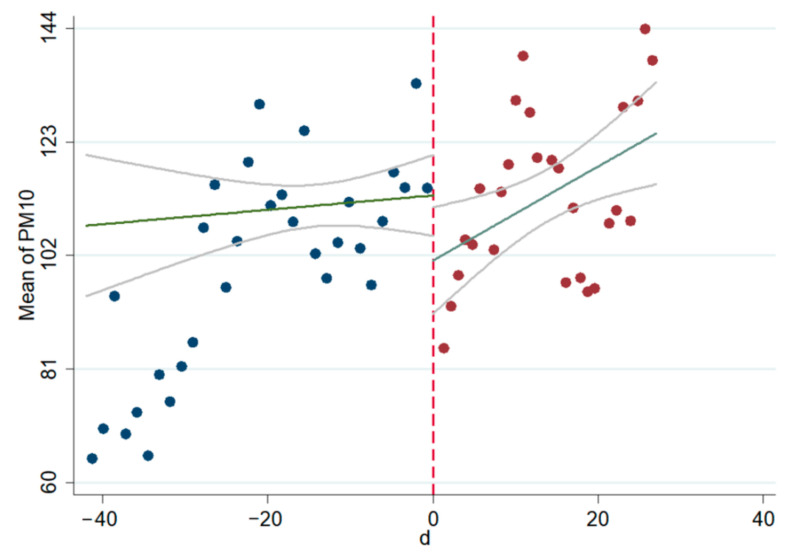
Scatter diagram of PM_10_ before and after the interviews (linear).

**Figure 8:**
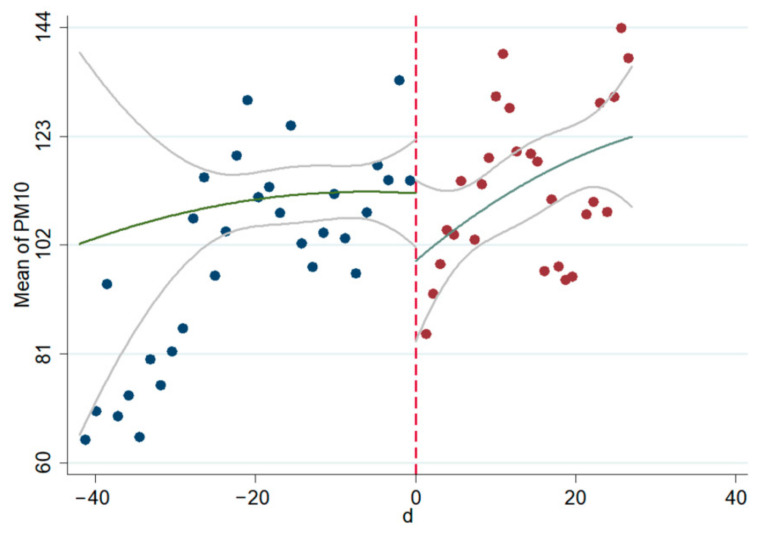
Scatter diagram of PM_10_ before and after the interviews (nonlinear).

The linear and non-linear scatter plots of AQI, PM_2.5_, and PM_10_ before and after the interviews during 2014–2020 are presented in Figure 3, Figure 4, Figure 5, Figure 6, Figure 7, Figure 8. The vertical axis depicts the concentration values of air quality indicators (including AQI, PM_2.5_, and PM_10_) and the horizontal axis depicts the time (four weeks before and after the interview). The “linear” line reflects the actual value. The lines beside the “linear” line reflect the confidence interval, showing the degree that the real value of this parameter has a certain probability to fall around the measurement result. The scatter plots show that AQI significantly improves after the interviews. As seen in Figure 3, Figure 4, Figure 5, Figure 6, Figure 7, Figure 8, AQI improves, and PM_2.5_ and PM_10_ concentration values are significantly lower after the interviews compared to before the interviews.

Based on the short-term effects of the full sample, the next step is to subdivide the sample into three for comparison (considering the factors that affect air quality): (1) interviewing cities with air problems versus interviewing cities without air problems; (2) heating cities versus non-heating cities; and (3) cities that are in the middle of heating and non-heating cities. The results of the analyses of subsamples are shown in Table 5, Table 6, Table 7.

Table 5 shows the results of regression analyses for interviewing cities without air problems and interviewing cities with air problems, and they are consistent with the estimation results of the full samples. The results show that the interview system has a significant effect on the reduction of PM_2.5_ and PM_10_ with some effect on the reduction of NO_2_. However, the effect on SO_2_, CO, and O_3_ is less than satisfactory. Air quality improvement in cities without air problems is slightly better than that of cities with air problems.

Table 6 shows the results of regression analyses for heating cities and non-heating cities, and they are consistent with the results for the full samples. The results show that PM_2.5_ and PM_10_ exhibit the most significant emission reduction, whereas NO_2_ emission reduction also has a considerable effect. At the same time, reductions in SO_2_ and CO emissions are not significant. The emission reduction of O_3_ in non-heating cities is better than that in heating cities.

Table 7 shows the results of regression analyses of heating and non-heating cities. The results are consistent with the results of the whole sample. The results show that emission reduction of PM_2.5_ and PM_10_ is the most significant, whereas that of SO_2_, NO_2_, CO, and O_3_ is not satisfactory.

### 3.4. Analysis of Long-Term Effects

To control the total sample size, daily frequency data require a smaller period and, thus, can only reflect the short-term effect. On the other hand, the weekly frequency data require a longer period and can reflect the long-term effect. This paper uses the weekly average post data to show the variation before and after the interview to illustrate the changes over a longer period to explore the long-term effects of the environmental interview system.

To ensure that the sample size of the weekly frequency is consistent with that of the daily frequency, the sample size was expanded to 17,472 in Section 3.4 and the data interval was set to 26 weeks before and after the interviews. Table 8 shows the RD estimation results after the weekly frequency treatment, where f(d) was selected based on the optimal first and second order equations for inclusion in the model. Concurrently, the estimated results are negative but insignificant, indicating that the interviews do not have long-term effects. This shows that in the implementation of China’s environmental interview system, the phenomenon of emergency short-term governance exists, and therefore, the governance effect is not long-term and sustainable.

## 4. Robustness Tests

In the previous analysis, we concluded that the air quality of the cities improved significantly in the short-term period after the environmental interviews even though the long-term effect was unsatisfactory. To make the results more reliable and credible, a robustness test was conducted.

### 4.1. Control Group Analysis

To prove that the changes are caused by the effect of the environmental interview system and are not sudden changes that were caused by other regulatory systems or national environmental protection actions, using an RD model, we can find cities with air conditions closest to those of the experimental group (before the environmental interview) and estimate whether there is an improvement before and after the interviews. 

The control group was considered the sample object, and the RD was estimated using dummy control variables of the heating city, undergoing heating, and working day. Table 9 shows that AQI, PM_2.5_, PM_10_, SO_2_, NO_2_, CO, and O_3_ of the control group are all insignificant (both positive and negative), indicating that the change in air quality after the control group’s interview was a normal fluctuation and not a significant improvement. The deterrent effect of the environmental interview system indicates that it improves the air quality only in those cities that were interviewed and also confirms that the improvement in air quality before and after the control group interviews was not a policy factor but a time effect of non-policy factors.

### 4.2. Sensitivity Analysis of Bandwidth

In the case of a certain number of cities being interviewed by the MEP, we chose different time intervals to test the stability of the model to eliminate the influence of different time frames on the estimation results.

Lee et al. [38] pointed out that different bandwidth settings for RD may affect the robustness of the results. Therefore, we used data from 2, 4, 6, and 8 weeks (before and after the interview) for the RD analysis. The results in Table 10 show that AQI, PM_2.5_, PM_10_, and NO_2_ are significantly negative for different bandwidths, whereas SO_2_, CO, and O_3_ are not significantly negative. This indicates that the estimation results are independent of bandwidth choice and the air quality improves significantly after the interview.

### 4.3. Validation of Air Quality

Governments may artificially fabricate air quality data to meet assessment targets as quickly as possible. Usually, the assessment is reported as compliance, secondary standard, good, “blue sky”, etc., so that we can remove the samples near these standards and check whether the remaining samples still show significant improvement.

Ghanem et al. [39] found that China’s air quality improved significantly after the adoption of the new air quality standards. However, to achieve the AQI assessment goal, the government may falsify data. We must carry out a robustness check on the authenticity of data. Based on the above analysis, we rejected data of AQI in three sections, namely, 95–100, 90–100, and 80–100, which were different from sections used by the other scholars (95–105, 90–110, and 80–120). It is meaningless to reject data above 100 because it is possible to falsify data to make it appear closer to ‘good data’, i.e., data that meet ideal standards.

Table 11 shows that the RD results are still significantly negative after removing the falsification prone intervals of AQI. Therefore, we conclude that after the environmental interviews, the government implemented a series of emission reduction measures to improve the air quality, rather than falsifying data.

## 5. Discussion

In practical terms, the current smog phenomenon in China is still relatively serious, frequently causing people to have respiratory symptoms, such as excessive sputum. Some cities are suffering from air quality problems that lead to migration and even brain drain. Air quality also affects the health of the people and the development of the national economy [40,41]. Some scholars believe that it may affect the stock market, the performances of companies, and so on [42,43]. At the theoretical level, the basis for policy evaluation has been relatively rich, but a review of the literature shows that environmental policies are not sufficiently evaluated [44,45]. Although the interview system has been applied in many fields, research on it has been qualitative. Furthermore, studies on the effects of the environmental interview system on air governance from the perspective of policy evaluation are rare [46].

**Figure 9:**
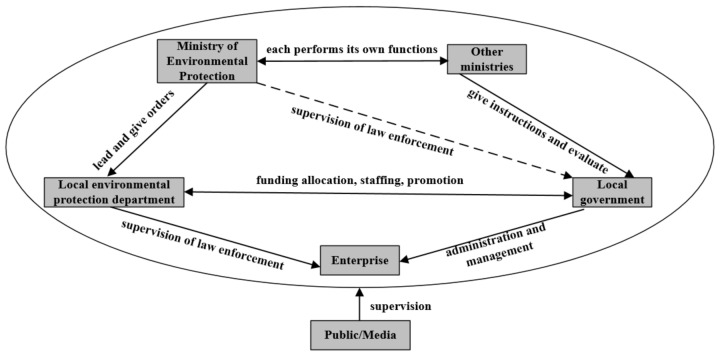
Environmental governance system.

The Ministry of Environmental Protection, taking environmental protection talks as the carrier, strives to transmit environmental protection pressure to local governments through the central administrative departments. Then the local governments transmit environmental pressure to local functional departments and enterprises, to fully activate the administrative resources of local governments through the linkage of the central, provincial, municipal, and county environmental protection negotiation system. It promotes the comprehensive improvement, prevention and control, and source treatment of the environment, promote the “interaction between the central government and the local government, the coordination of various departments, and the same responsibility of the party and the government” in environmental protection. It realizes the unification of the central ecological environmental protection policy and the local economic development goals. The environmental governance system is shown in Figure 9.

The central government monitors local governments through a ‘cross-level’ dialogue and consultation mechanism, effectively promoting the implementation of policies. This paper provides an in-depth analysis of the role of environmental interview systems on the impact of air governance and proposes a new method for determining the breakpoint, thereby improving the assessment of its long-term effects by subdividing different samples. Therefore, this study not only enriches the theories related to environmental interview systems but also provides revised model treatments and provides multidimensional research perspectives for future scholarly research.

**Figure 10:**
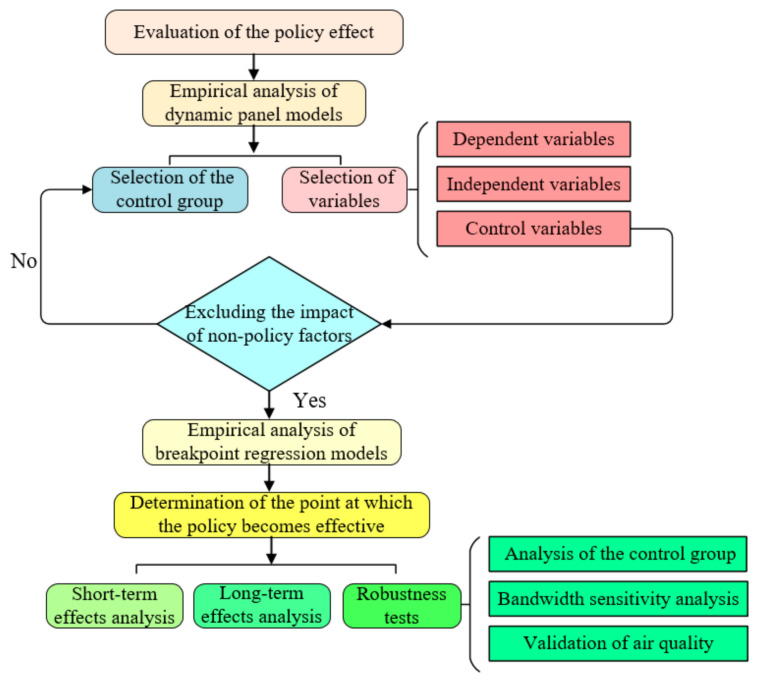
The process of evaluating the policy effectiveness of the environmental interview system.

The process of evaluating the policy effectiveness of the environmental interview system is shown as follows (Figure 10):

The theoretical and empirical analyses in this paper provide in-depth analyses. However, some other problems were not addressed in the present study due to the limitation of space. The following points can be considered in future studies:

(1) The environmental interview system is too complex to quantify every aspect and throw into a regression equation. Other possible explanations for the observed changes should be considered, explored, and addressed in the future.
(2) The sample can be subdivided to accommodate multiple perspectives to carefully consider the factors influencing policy transmission; more angles can be used for comparative analysis.
(3) The choice of cities in the control group is crucial. It needs to be comprehensively considered from the aspects of population statistics, political system, political factors, and dependent variables. More control variables can be added, and the processing of variables can be more quantitative and refined to obtain assessment results with higher accuracy [47].

## 6. Conclusions

The effectiveness of the government environmental policy is the key to affect the quality of the environment, which provides a reference for further policy design [48]. In this paper, we provided details on air pollution control in the interviewed cities and their surrounding cities. Furthermore, 48 cities that were publicly interviewed from 2014–2020 were selected as the experimental group and 48 surrounding cities as the control group. We applied the dynamic panel model to initially judge the policy effect, and then, used the sharp RD method to assess the short-term and long-term effects of the environmental interview system and analyzed the reasons for the differences between the estimates of different types of samples. Finally, we conducted a robustness test from multiple perspectives.

Based on the above analysis, the following results can be drawn:

(1) The regression discontinuity (RD) method, for both the whole sample and subsample, shows that the implementation of the environmental protection interview system improved air quality. Concentrations PM_2.5_ and PM_10_ were significantly reduced and were consistent in terms of environmental protection performance.
(2) RD analysis of the long-term sustained effect of the interview system conducted using weekly average data showed air quality improvement and reduction in the concentrations of PM_2.5_ and PM_10_. However, the effect was not significant.
(3) By removing likely ranges in which data falsification can occur the control group was analyzed using bandwidth sensitivity test, and the validation RD analysis proved that the interview system can improve air quality by ruling out data falsification.

We evaluated air quality governance using China’s environmental protection interview system and provide an in-depth analysis of its mechanism. The results show that the environmental protection interview system can indeed improve air quality, but it has an emergency short-term influence and therefore it is not long-term and sustainable. Assesses the effect of the environmental interview system to improve air quality from a multi-dimensional perspective through long- and short-term comparisons. Improved evaluation methods are used to obtain comprehensive and objective evaluation results, which can provide timely feedback on policy effects and help analyze policy deviations. Although the scope of the research is regional, it has important implications for the world, especially in countries with similar economic systems that face a trade-off between economic growth and environmental sustainability [49]. The results of this study will serve as a reference for further action by the government.

## Figures and Tables

**Table 1:** Symbols and meanings of various variables.

	Variable Symbol	Unit	Meaning
Dependent variables	AQI	Index	It is the air quality index while considering GB3095-2012 * ambient air quality standard (current), as the reference standard, and the concentration of pollutants (SO_2_, NO_2_, PM_10_, PM_2.5_, O_3_, and CO); their corresponding indices are published once every hour.
	PM_2.5_	μg/m^3^	It is the fine particulate matter, aerodynamically less than or equal to 2.5 microns in diameter; it is divided into natural sources (such as dust) and anthropogenic sources (such as primary and secondary particulate matter).
	PM_10_	μg/m^3^	It is a respirable particulate matter, with an aerodynamic diameter of less than or equal to 10 microns.
	SO_2_	μg/m^3^	The concentration of sulfur dioxide.
	NO_2_	μg/m^3^	The concentration of nitrogen dioxide.
	CO	mg/m^3^	The concentration of carbon monoxide.
	O_3_	μg/m^3^	The concentration of ozone.
Independent variables	T	Dummy variable	It is also known as the driver variable, which is 0 before the interview and 1 after the interview; it indicates the policy treatment effect in RD.
	d	Days or weeks	It indicates the number of days or weeks from the date of the interview; negative values imply pre-interview; other values imply post-interview.
	f(d)		It is a polynomial function with “d” as the independent variable, i.e., air quality improvement resulting from the gradual advancement of pollution prevention efforts.
Control variables	I	Dummy variable	It is 1 if there is public heating; otherwise, it is 0.
	J	Dummy variable	It is 1 if the sample is in the heating period; otherwise, it is 0.
	K	Dummy variable	It is 1 if the day is a working day; otherwise, it is 0.
	YU	Dummy variable	It is 1 if it has rained on that day but not yet snowed; 0 if it has not yet rained.
	XUE	Dummy variable	It is 1 if it has snowed on that day; otherwise, it is 0.
	MAXT	°C	The highest temperature of the day.
	MINT	°C	The lowest temperature of the day.
	MAXW	Level	Maximum wind strength for the day.

* Note: Gb3095-2012 is the ambient air quality standard of the people’s Republic of China, issued by the Ministry of environmental protection of the people’s Republic of China in 2012. This standard applies to the assessment and management of ambient air quality.

**Table 2:** Dynamic panel fixed effects of LSDV estimation results.

The Dependent Variable	AQI	PM_2.5_	PM_10_	SO_2_	NO_2_	CO	O_3_
T	−3.49 **	−3.38 **	−4.308 **	−2.038 **	−0.948 **	−0.06 ***	1.73 ***
YU	−6.93 ***	−3.03 *	−9.85 ***	−3.10 ***	−1.75 ***	−0.01	−8.95 ***
XUE	−3.44	−5.38	−5.47	−8.72 ***	−1.51	−0.04	−1.25
MAXT	1.31 ***	0.66 **	2.16 ***	0.31	0.43 ***	0.01 **	0.84 ***
MINT	−1.00 ***	−0.51 *	−1.70 ***	−0.59 ***	−0.58 ***	−0.01 ***	−0.01
MAXW	−7.41 ***	−9.86 ***	−6.64 ***	−6.86 ***	−4.96 ***	−0.13 ***	1.93 ***
Lag of first order	0.58 ***	0.61 ***	0.54 ***	0.70 ***	0.56 ***	0.57 ***	0.61 ***
Constant term	55.08 ***	54.24 ***	53.20 ***	36.24 ***	31.35 ***	0.98 ***	4.26 *
(1)	Yes	Yes	Yes	Yes	Yes	Yes	Yes
(2)	Yes	Yes	Yes	Yes	Yes	Yes	Yes
(3)	Yes	Yes	Yes	Yes	Yes	Yes	Yes
Sample size	5712	5712	5712	5712	5712	5712	5712
Goodness of fit	0.4866	0.5053	0.4581	0.7877	0.622	0.6122	0.7223

Note: *** indicates that the variable is significant at the 0.1% significance level, ** indicates that the variable is significant at the 1% significance level, * indicates that the variable is significant at the 5% significance level; (1) represents whether heating versus non-heating cities was considered; (2) represents whether heating was being considered; (3) represents whether working days versus non-working days were considered; data were analyzed by Stata 14.0.

**Table 3:** List of urban air quality improvement time-points.

Region	T	Significant or Not	Region	T	Significant or Not	Region	T	Significant or Not
Shenyang	0	Yes	Lvliang	1	Yes	Beijing	6	No
Kunming	3	Yes	Ma’anshan	7	Yes	Tianjin	3	Yes
Changchun	2	Yes	Xingtai	1	No	Shijiazhuang	8	Yes
Zhumadian	5	No	Zhengzhou	0	Yes	Tangshan	6	No
Baoding	1	No #	Nanyang	7	Yes	Handan	8	Yes
Wuxi	6	Yes	Baise	0	Yes	Hengshui	8	Yes
Ziyang	1	Yes	Haixi	3	Yes	Yuncheng	0	Yes
Zhangye	7	Yes	Dezhou	1	No	Tianjin ②	1	No
Siping	5	Yes	Jining	10	Yes	Handan ②	5	Yes
Jingdezhen	8	Yes	Shangqiu	2	No	Baoding ②	1	Yes
Zibo	2	Yes	Anqing	3	Yes	Xinxiang	5	Yes
Anyang	6	Yes	Changzhou	2	Yes	Harbin ②	7	No
Harbin	0	No	Xianyang	3	No	Jimujia	0	Yes
Cangzhou	0	No #	Yangquan	0	Yes	Shuangyashan	0	Yes
Linyi	2	No #	Weinan	3	No	Hegang	10	No
Chengde	0	Yes	Linfen	0	Yes	Linfen ②	0	Yes

Note: ② indicates that the city was interviewed for the second time and the interview included air issues; the city interviewed for the second time is considered here as ‘another city’. *p* < 0.05 is considered significant; # represents deterioration; lagged days were obtained from Stata 14.0 analysis.

**Table 4:** Regression discontinuity (RD) estimation results of full sample daily frequency.

Dependent Variable	AQI	PM_2.5_	PM_10_	SO_2_	NO_2_	CO	O_3_
First order	−46.46 ***	−42.51 ***	−52.12 ***	−7.40	−8.33 **	−0.35	−0.82
	8.8291	8.3258	10.5334	5.9949	2.8363	0.2237	3.9625
Second order	−48.53 ***	−44.06 **	−54.91 **	−5.23	−9.82 *	−0.20	−0.06
	14.9662	14.2437	17.7622	9.2229	4.7775	0.5901	6.1667
(1)	Yes	Yes	Yes	Yes	Yes	Yes	Yes
(2)	Yes	Yes	Yes	Yes	Yes	Yes	Yes
(3)	Yes	Yes	Yes	Yes	Yes	Yes	Yes
Sample size (N)	2688	2688	2688	2688	2688	2688	2688

Note: *** indicates that the variable is significant at the 0.1% significance level, ** indicates that the variable is significant at the 1% significance level, * indicates that the variable is significant at the 5% significance level.

**Table 5:** Results of RD estimation of the daily frequency of interviewing cities with air problems versus interviewing cities without air problems.

The Dependent Variable	AQI	PM_2.5_	PM_10_	SO_2_	NO_2_	CO	O_3_
Excluding airborne causes	−45.45 ***	−37.08 ***	−54.91 ***	−18.08 **	−10.54 **	−0.54	−9.43
N = 616	11.7606	10.6945	13.1813	6.4863	3.5994	0.3463	5.3983
Includes airborne causes	−43.58 ***	−41.06 ***	−47.55 ***	−5.20	−7.17 *	−0.22	1.04
N = 2072	10.0222	9.2230	12.5126	7.0135	3.2058	0.2567	4.0487
(1)	Yes	Yes	Yes	Yes	Yes	Yes	Yes
(2)	Yes	Yes	Yes	Yes	Yes	Yes	Yes
(3)	Yes	Yes	Yes	Yes	Yes	Yes	Yes
Time trend items	1st order	1st order	1st order	1st order	1st order	1st order	1st order

Note: *** indicates that the variable is significant at the 0.1% significance level, ** indicates that the variable is significant at the 1% significance level, * indicates that the variable is significant at the 5% significance level.

**Table 6:** Results of RD estimation of daily frequency in heated and non-heated cities.

The Dependent Variable	AQI	PM_2.5_	PM_10_	SO_2_	NO_2_	CO	O_3_
Heating cities	−48.98 ***	−44.61 ***	−55.02 ***	−9.72	−9.31 **	−0.42	1.82
N = 2184	10.1897	9.3674	12.6496	7.0766	3.1568	0.2665	4.0645
Non-heated cities	−36.12 ***	−30.78 ***	−41.47 ***	−2.28	−6.77 *	−0.03	−14.71 *
N = 504	6.7566	6.1264	10.6002	2.9051	3.5847	0.1861	6.1101
(2)	Yes	Yes	Yes	Yes	Yes	Yes	Yes
(3)	Yes	Yes	Yes	Yes	Yes	Yes	Yes
Time trend items	First order	First order	First order	First order	First order	First order	First order

Note: *** indicates that the variable is significant at the 0.1% significance level, ** indicates that the variable is significant at the 1% significance level, * indicates that the variable is significant at the 5% significance level.

**Table 7:** Results of RD estimation of daily frequency in heating versus non-heating cities.

The Dependent Variable	AQI	PM_2.5_	PM_10_	SO_2_	NO_2_	CO	O_3_
Heating	−79.76 ***	−78.47 ***	−90.95 ***	−21.39	−18.05 **	−1.02 *	6.12
N = 896	21.3759	21.3534	25.2649	17.6944	5.8145	0.4556	4.4694
Non-heated	−28.99 ***	−25.13 ***	−31.56 ***	1.77	−4.00	−0.04	−3.89
N = 1792	5.1775	4.8046	7.0471	2.3667	2.4217	0.1602	4.1979
(1)	Yes	Yes	Yes	Yes	Yes	Yes	Yes
(3)	Yes	Yes	Yes	Yes	Yes	Yes	Yes
Time trend items	1st order	1st order	1st order	1st order	1st order	1st order	1st order

Note: *** indicates that the variable is significant at the 0.1% significance level, ** indicates that the variable is significant at the 1% significance level, * indicates that the variable is significant at the 5% significance level.

**Table 8:** Results of RD estimation of daily frequency in heating vs. non-heating cities.

Dependent Variable	AQI	PM_2.5_	PM_10_	SO_2_	NO_2_	CO	O_3_
First order	9.9954	8.4567	7.6231	−1.4474	1.9527	0.0732	2.6421
	5.2740	5.1546	6.3249	5.9426	2.1686	0.1578	3.4661
Second order	9.9568	8.7332	9.6521	−1.8060	2.1293	0.1427	1.4919
	7.6928	7.9641	9.1196	9.1845	3.3072	0.4084	5.2213
(1)	Yes	Yes	Yes	Yes	Yes	Yes	Yes
(2)	Yes	Yes	Yes	Yes	Yes	Yes	Yes
(3)	Yes	Yes	Yes	Yes	Yes	Yes	Yes
Sample size (N)	17,472	17,472	17,472	17,472	17,472	17,472	17,472

**Table 9:** Results of daily frequency RD estimation for the control group.

Dependent Variable	AQI	PM_2.5_	PM_10_	SO_2_	NO_2_	CO	O_3_
First order	−3.4414	0.8399	−1.3651	1.5155	3.9088	0.1210	−1.7716
	7.0512	6.2666	8.3059	4.1755	2.9094	0.1933	4.7489
Second order	−13.4233	−5.1951	−7.3566	2.6986	3.0253	0.4285	−4.9183
	10.9033	9.4684	12.7148	6.3938	4.2867	0.3885	7.2495
(1)	Yes	Yes	Yes	Yes	Yes	Yes	Yes
(2)	Yes	Yes	Yes	Yes	Yes	Yes	Yes
(3)	Yes	Yes	Yes	Yes	Yes	Yes	Yes
Sample size (N)	2688	2688	2688	2688	2688	2688	2688

**Table 10:** Estimated results for different bandwidths.

Dependent Variable	2 Weeks before and after the Interview	4 Weeks before and after the Interview	6 Weeks before and after the Interview	8 Weeks before and after the Interview
AQI	−48.58505 ***	−47.10203 ***	−48.6484 ***	−48.46691 ***
PM_2.5_	−44.81683 ***	−43.13503 ***	−45.12565 ***	−44.98694 ***
PM_10_	−53.16457 ***	−52.46213 ***	−53.90612 ***	−53.68375 ***
SO_2_	−53.16457 ***	−7.226675	−6.67132	−6.681053
NO_2_	−8.765467 **	−8.307712 **	−8.775167 **	−8.73947 **
CO	−0.35734	−0.362782	−0.355508	−0.353217
O_3_	−1.226568	−1.387607	−1.043695	−1.079751
Sample size (N)	1344	2688	4032	5376

Note: *** indicates that the variable is significant at the 0.1% significance level, ** indicates that the variable is significant at the 1% significance level.

**Table 11:** Estimated results after removing potentially falsified data.

Dependent Variable		Excluding (95–100)	Excluding (90–100)	Excluding (80–100)
AQI	First order	−50.14 ***	−52.21 ***	−61.39 ***
		9.5744	10.0546	12.1070
	Second order	−53.29 ***	−54.48 ***	−67.26 **
		16.2056	16.9812	21.1922
PM_2.5_	First order	−45.98 ***	−47.45 ***	−54.59 ***
		9.0523	9.4447	11.0715
	Second order	−49.47 ***	−50.37 **	−61.05 **
		15.4643	16.0838	19.5383
PM_10_	First order	−56.32 ***	−58.82 ***	−70.26 ***
		11.5673	12.1509	14.4111
	Second order	−61.23 **	−62.25 **	−79.07 **
		19.5845	20.5753	25.1163
Sample size (N)		2566	2422	2158

Note: *** indicates that the variable is significant at the 0.1% significance level, ** indicates that the variable is significant at the 1% significance level.

## Data Availability

The data were mainly collected from the China City Statistical Yearbooks and the database of the CNRDS platform (https://www.cnrds.com/ accessed on 11 May 2014).

## Appendix A

**Table A1:** MEP interviewed cities (experimental group) and control group (2014–2020).

	Experimental Group	Control Group	(a)	(b)
1	Shenyang	Fushun	Yes	Yes
2	Kunming	Yuxi	No	No
3	Changchun	Jilin	Yes	Yes
4	Zhumadian	Luohe	No	No
5	Baoding	Shijiazhuang	Yes	No
6	Wuxi	Changzhou	No	No
7	Ziyang	Neijiang	No	No
8	Zhangye	Jinchang	Yes	No
9	Siping	Changchun	Yes	No
10	Jingdezhen	Nanchang	No	No
11	Zibo	Ji’nan	Yes	No
12	Anyang	Hebi	Yes	Yes
13	Harbin	Daqing	Yes	Yes
14	Cangzhou	Tianjin	Yes	Yes
15	Linyi	Rizhao	Yes	Yes
16	Chengde	Langfang	Yes	Yes
17	Lvliang	Taiyuan	Yes	No
18	Ma’anshan	Wuhu	No	No
19	Xingtai	Shijiazhuang	Yes	No
20	Zhengzhou	Shijiazhuang	Yes	No
21	Nanyang	Pingdingshan	No	No
22	Baise	Hechi	No	No
23	Haixi	Hainan	Yes	Yes
24	Dezhou	Liaocheng	Yes	Yes
25	Jining	Tai’an	Yes	No
26	Shangqiu	Zhoukou	Yes	No
27	Anqing	Chizhou	No	No
28	Changzhou	Jincheng	Yes	No
29	Xianyang	Xi’an	Yes	No
30	Yangquan	Taiyuan	Yes	Yes
31	Weinan	Xi’an	Yes	Yes
32	Linfen	Yuncheng	Yes	Yes
33	Beijing	Langfang	Yes	No
34	Tianjin	Langfang	Yes	No
35	Shijiazhuang	Xingtai	Yes	No
36	Tangshan	Tianjin	Yes	No
37	Handan	Xingtai	Yes	No
38	Hengshui	Cangzhou	Yes	No
39	Yuncheng	Jincheng	Yes	No
40	Tianjin②	Langfang	Yes	No
41	Handan ②	Langfang	Yes	No
42	Baoding ②	Shijiazhuang	Yes	No
43	Xinxiang	Jiaozuo	Yes	No
44	Harbin ②	Suihua	Yes	Yes
45	Jiamusi	Qitaihe	Yes	Yes
46	Shuangyashan	Jixi	Yes	Yes
47	Hegang	Yichun	Yes	Yes
48	Linfen ②	Yuncheng	Yes	No

Note: ② indicates that the city was interviewed for the second time and the interview included air issues; the city interviewed for the second time is considered here as ‘another city’. (a) indicates whether the city is being heated; (b) indicates whether the city is in the process of being heated.
